# Congenital rubella syndrome exposure in a pediatric hospital: experience from developing world

**DOI:** 10.1186/2047-2994-4-S1-P260

**Published:** 2015-06-16

**Authors:** D Sureshkumar, R Gopalakrishnan, L Jessani

**Affiliations:** 1 Infectious Disease, Apollo Hospitals, Chennai, India

## Introduction

Congenital rubella syndrome (CRS) is a rare disorder with devastating ocular and systemic consequences. Although efforts to eradicate the disease have been in place for some time, developing world continue to be affected by this disease

## Objectives

We describe an exposure to a six month old infant with congenital rubella syndrome (CRS) in the pediatric hospital and the successful management of exposure and prevention of rubella cases among exposed staff with limited resources.

## Methods

In January 2015, a six month old infant with a patent ductus arteriosus was admitted to a 50 bedded tertiary referral pediatric hospital in South India. The infant was seen & managed by cardiologist initially followed by cardio thoracic surgery team, pediatricians, intensivists and ophthalmologist. Although CRS was mentioned in the differential diagnoses of several physicians, the patient was not isolated until the 8th day of hospitalization. This may be part due to the unrecognized nature & duration of infectivity of CRS.

## Results

Totally thirty five health care workers (HCWs) exposed to CRS child during his initial 8 days of stay in the hospital. Majority of the exposure happened during his stay in multi bedded general ward (18/35), followed by cardio thoracic intensive care unit (4/35) and in the operating room (3/35). Two of the exposed employees were pregnant that time. An attempt was made to prevent an outbreak of additional exposures by the isolation (both contact & droplet precautions) of the infant, rubella Ig G antibody testing of both pregnant health care workers with unknown or uncertain history of rubella vaccination (both were immune) and prompt administration of rubella vaccine to significantly exposed persons.

## Conclusion

Outbreaks of CRS that occur in pediatric hospitals in the developing world are of special concern. Testing of all employees for rubella antibody and immunization of those determined to be seronegative should be considered. Infants with congenital rubella syndrome shed rubella virus in large quantity for prolonged periods in urine and saliva. They need to be kept in both contact & droplet isolation for minimum 1 year to avoid unprotected exposures among HCWs

## Disclosure of interest

None declared.

